# Engineering the Dipole Orientation and Symmetry Breaking with Mixed‐Dimensional Heterostructures

**DOI:** 10.1002/advs.202200082

**Published:** 2022-05-09

**Authors:** Md Gius Uddin, Susobhan Das, Abde Mayeen Shafi, Vladislav Khayrudinov, Faisal Ahmed, Henry Fernandez, Luojun Du, Harri Lipsanen, Zhipei Sun

**Affiliations:** ^1^ Department of Electronics and Nanoengineering Aalto University Tietotie 3 Espoo FI‐02150 Finland; ^2^ QTF Centre of Excellence Department of Applied Physics Aalto University Aalto FI‐00076 Finland

**Keywords:** 2D materials, dipole engineering, mixed‐dimensional heterostructures, nanowires, optical anisotropy, symmetry breaking

## Abstract

Engineering of the dipole and the symmetry of materials plays an important role in fundamental research and technical applications. Here, a novel morphological manipulation strategy to engineer the dipole orientation and symmetry of 2D layered materials by integrating them with 1D nanowires (NWs) is reported. This 2D InSe –1D AlGaAs NW heterostructure example shows that the in‐plane dipole moments in InSe can be engineered in the mixed‐dimensional heterostructure to significantly enhance linear and nonlinear optical responses (e.g., photoluminescence, Raman, and second harmonic generation) with an enhancement factor of up to ≈12. Further, the 1D NW can break the threefold rotational symmetry of 2D InSe, leading to a strong optical anisotropy of up to ≈65%. These results of engineering dipole orientation and symmetry breaking with the mixed‐dimensional heterostructures open a new path for photonic and optoelectronic applications.

## Graphical abstract

2D InSe‐1D AlGaAs nanowire mixed‐dimensional heterostructures offer engineered in‐plane dipole moments and threefold rotational symmetry breaking in InSe, resulting in significantly enhanced optical responses with strong optical anisotropy.

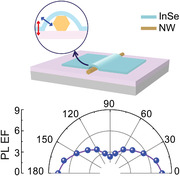

## 1 Introduction

Engineering the dipole and the symmetry of materials is important in modifying their macroscopic electrical, optical, magnetic, and topological properties.^[^ ^1^ ^]^ Over the past few years, a wide variety of approaches have been developed to engineer the dipole and symmetries of two‐dimensional (2D) materials with twist,^[^ ^2^ ^]^ strain,^[^ ^3^ ^]^ external electric,^[^ ^4^ ^]^ and magnetic^[^ ^5^ ^]^ approaches. These strategies offer unprecedented possibilities to manipulate the internal quantum degrees of freedom in 2D materials and, therefore, provide a firm basis for various new physical phenomena, for example, intriguing anisotropies in vibrational and optical properties,^[^ ^6^ ^]^ highly tunable second‐order nonlinear optical responses,^[^ ^7^ ^]^ and exotic spin‐orbit physics.^[^ ^8^ ^]^

Recently, 2D laminar InSe has aroused tremendous attention owing to its exceptional optical, electronic, and mechanical properties.^[^ ^9^, ^10^, ^11^ ^]^ It exhibits excellent electron mobility (≈10^3^ cm^2^ V^−1^s^−1^)^[^ ^12^ ^]^ due to the small effective mass of electrons and weak electron‐phonon scattering.^[^ ^13^, ^14^ ^]^ It also shows layer‐dependent bandgap spanning from the visible to the near‐infrared region for numerous applications, for example, photodetection^[^ ^15^, ^16^ ^]^ and sensing.^[^ ^17^ ^]^ Interestingly, it shows indirect‐to‐direct band‐gap crossover upon increasing thickness.^[^ ^9^, ^18^ ^]^ Additionally, the orbital nature of the band edges of InSe is distinct from traditional transition metal dichalcogenides (TMDCs). In particular, the conduction band minimum of InSe arises from s orbital of In atom, whereas the top valence band maximum is dominated by the p*_z_* orbital of Se atom.^[^ ^19^, ^20^, ^21^ ^]^ Consequently, in stark contrast to the widely studied TMDCs, the absorption dipole of InSe is out‐of‐plane (OP). Exciton transitions are fully allowed only for light polarization E||c, where c is the direction normal to the InSe layers.^[^ ^22^, ^23^, ^24^, ^25^, ^26^, ^27^ ^]^ The transitions with E⊥c are only weakly allowed through a weak spin‐orbit interaction involving holes in the deeper valence bands with Se p*_x_* and p*_y_* orbitals.^[^ ^28^, ^29^, ^30^ ^]^ Therefore, unlike typical TMDCs with in‐plane (IP) dipole, the band edge transitions of InSe cannot couple effectively to light at a normal incident angle, largely hindering the InSe‐based potential optoelectronic applications. Different approaches have been demonstrated to engineer the dipole orientation of InSe and enhance the light–matter interactions, for example, by applying strain,^[^ ^31^ ^]^ nanotexturing with SiO_2_ nanoparticles,^[^ ^32^ ^]^ bending the flake onto an array of Si‐nanopillars,^[^ ^33^ ^]^ and forming localized wrinkles.^[^ ^34^ ^]^

In this work, we report a new approach to engineer the dipole orientation of InSe by integrating 2D InSe with 1D AlGaAs nanowire (NW). Benefitting from the efficient change of the intrinsic OP dipole moments into IP moments, both linear and nonlinear optical responses (e.g., Raman, photoluminescence (PL), and second harmonic generation (SHG)) of InSe are significantly enhanced in the mixed‐dimensional heterostructures. Remarkably, the mixed‐dimensional heterostructures can break the threefold rotational symmetry of 2D InSe, leading to strong anisotropic linear and nonlinear optical responses. The engineering of both dipole orientation and symmetry with mixed‐dimensional structure paves a new way for various optoelectronic applications.

## 2 Results and Discussion

**Figure 1:**
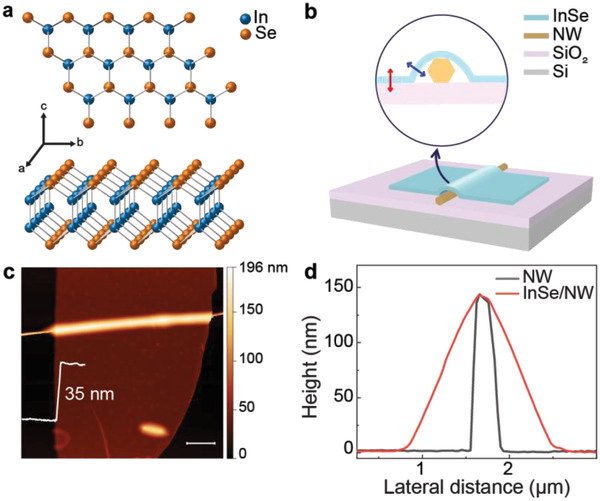
Engineering of dipole orientation of InSe with a 2D–1D mixed‐dimensional heterostructure. a) Crystal structures of InSe viewed from the *c*‐ (top) and the *b*‐axis (bottom) directions. b) Schematic of an InSe‐NW mixed‐dimensional heterostructure. The zoom‐in view (inset) depicts the manipulation of the intrinsic out‐of‐plane dipole (red arrow) of InSe by taking advantage of the modified morphology of InSe owing to the presence of the NW underneath. c) AFM topography of a typical mixed‐dimensional heterostructure along with the thickness profile of the InSe flake. Scale bar: 3 µm. d) Comparison of the height profile of the NW and the InSe flake transferred onto it. Note that the height results shown in the figure do not represent the real profiles of the samples, as the unit in the *y*‐axis (nm) is different from that in the *x*‐axis (µm).

InSe (*γ* ‐phase) crystals have a honeycomb lattice structure, as depicted in **Figure** **1**a. In each layer, the crystal lattice consists of four covalently bonded atomic planes arranged in the sequence of Se‐In‐In‐Se. The key concept of this work, which deals with the engineering of dipole orientation and rotational‐symmetry breaking of InSe by integrating 2D InSe with 1D AlGaAs NW, are illustrated in Figure 1b. In the inset, the vertical red double‐headed arrow represents the intrinsic OP dipole moment of a bare InSe flake. NW modifies the topography of the flake in its vicinity and thus the dipole orientation, resulting in engineered IP dipole moments (presented with a blue tilted arrow). Our 2D InSe‐1D NW mixed‐dimensional van der Waals heterostructures are fabricated using the dry transfer technique (details in Experimental Section). Our experiments preferentially use multilayer InSe flakes as they have a brighter prospect in optoelectronic device applications than the few‐layer InSe flakes, which are not stable in ambient conditions.^[^ ^35^ ^]^ Figure 1c shows the atomic force microscopy (AFM) mapping result of a typical mixed‐dimensional heterostructure sample. The white line presented in the image depicts the height profile of the InSe flake, indicating that its thickness is ≈35 nm. Figure 1d shows the height profile of the 1D AlGaAs NW and the InSe/NW heterostructure. The results show that the diameter of the NW in the hybrid device is ≈140 nm.

**Figure 2:**
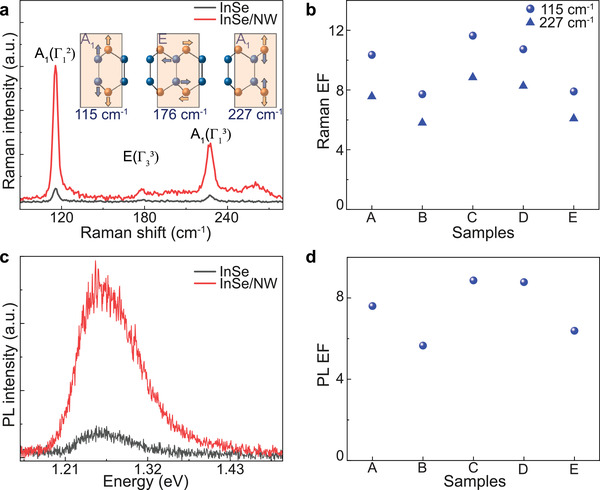
Enhanced optical properties of InSe with dipole orientation engineering. a) Comparison of Raman spectra of multilayer InSe with and without NW obtained with 532‐nm laser excitation at room temperature. b) Enhancement factors of Raman modes in different samples. c) Comparison of PL spectra under the same condition. d) PL enhancement factor in different samples.

In the mixed‐dimensional heterostructures, enhanced light–matter interactions are expected owing to the nonvanishing IP dipole moments. To confirm this, we perform both the linear and nonlinear optical measurements (e.g., Raman, PL, and SHG). **Figure** **2**a shows the Raman spectra of bare InSe (black line) and mixed‐dimensional heterostructures (red line) excited by a 532 nm laser at room temperature. Three distinctive Raman modes of InSe are observed in the range of 100–300 cm^−1^: A_1_(Γ) at ≈115 cm^−1^, E(Γ) at ≈176 cm^−1^, and A_1_(Γ) at ≈227 cm^−1^, being in good agreement with previous reports.^[^ ^31^, ^33^ ^]^ The origin of the three Raman modes is depicted in the inset. The peak at ≈260 cm^−1^ belongs to the AlGaAs NW.^[^ ^36^ ^]^ By comparing the InSe spectra with and without the NW, we find that the presence of NW underneath the InSe results in a dramatic intensity increase for all the three Raman modes of InSe. Compared with the previously reported results,^[^ ^31^ ^]^ our small Raman mode shift (a shift of −0.78 ± 0.1 cm^−1^ for the 176 cm^−1^ Raman mode, and a shift of −0.77 ± 0.1 cm^−1^ for the 227 cm^−1^ Raman mode) indicates that the strain enhanced Raman effect in our mixed‐heterostructures is negligible (Figure S1, Supporting Information).

We fabricate and test multiple samples and find reproducible results with relatively large diameter (>120 nm) NWs. Enhancement factor (EF), defined as the intensity ratio between the samples with and without NW, of the ≈115 and 227 cm^−1^ Raman modes obtained from five different mixed‐dimensional heterostructure samples are plotted in Figure 2b. The maximum EF is ≈12 with Sample C. In general, the average EFs of 5 samples at the ≈115 and 227 cm^−1^ modes are ≈9.7 and ≈7.3, respectively.

Figure 2c shows the typical PL spectra of InSe (black line) and mixed‐dimensional heterostructures (red line) under 532 nm excitation. In close resemblance to the Raman response, we observe a significant increase in the PL intensity of InSe from the mixed‐dimensional structures with a small peak shift (<≈9 meV) (Figure S2, Supporting Information). Compared with the previously reported results,^[^ ^37^ ^]^ our small PL peak shift indicates that the strain enhanced PL effect in our mixed‐dimensional heterostructures is negligible. Figure 2d represents PL EF calculated from different samples. The highest EF is ≈9, while the average from the five samples under study is ≈7.3.

Note that a similar enhancement effect in Raman and PL response is observed in mixed‐dimensional heterostructures with different NW diameters. In general, smaller diameter (<70 nm) NWs typically give a smaller PL EF of less than 2. This is reasonable due to the small bending angle with a small NW diameter. However, due to the fact that the bent InSe width cannot be fully controlled, we cannot fully correlate the EF as the function of the NW diameter. Further, we observe a similar PL enhancement of InSe when we replace the AlGaAs NW with an InP NW in the heterostructure. This shows that the enhancement in the mixed‐dimensional heterostructures is not dependent on the material of the NWs.

**Figure 3:**
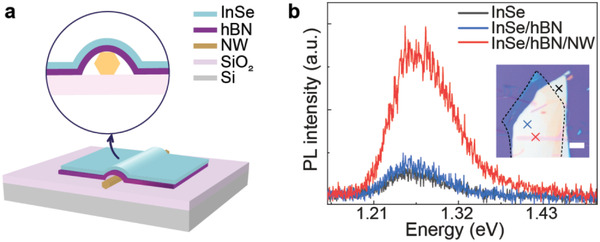
Dipole engineering in a mixed‐dimensional InSe/hBN/NW heterostructure sample. a) Schematic of the InSe/hBN/NW device. b) Comparison of PL spectra at different locations. Inset shows a corresponding optical image. The measurement positions are labeled in the image. The dashed line indicates the hBN flake. Scale bar: 5 µm.

To determine whether the largely enhanced Raman and PL responses of InSe/NW mixed‐dimensional heterostructures are from nonvanishing IP dipole moments or other reasons (e.g., charge transfer effects), we intercalate a 3‐nm thick hexagonal boron nitride (hBN) flake between InSe and NW as shown in **Figure** **3**a. If the enhanced Raman and PL responses are due to charge transfer effects, the thin hBN intercalation layer would block the charge transfer and disable the enhancement. Figure 3b shows the PL spectra of InSe (black line), InSe/hBN (blue line), and InSe/hBN/NW (red line). Interestingly, the InSe/hBN/NW structure also shows strong PL enhancement, ruling out the possibility of charge transfer. Further, we rule out optical interference, suspension, and energy transfer effects in the mixed‐dimensional heterostructures with a 40‐nm thick hBN flake between InSe and NW (Figure S3, Supporting Information). Therefore, the observed Raman and PL enhancement most likely comes from nonvanishing IP dipole moments and enhanced light–matter interactions.

**Figure 4:**
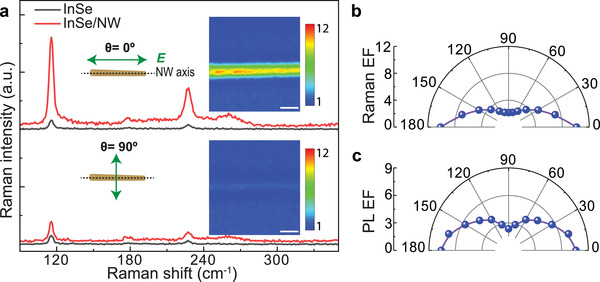
Broken‐symmetry induced anisotropic response from the mixed‐dimensional heterostructures. a) Comparison of Raman spectra when the excitation light polarization is parallel (*θ* = 0°) and perpendicular (*θ* = 90°) to the NW axis. The polarization direction of the linearly polarized excitation (green double‐arrow) is shown with an NW (NW axis is marked with a dotted line) in the middle inset. Corresponding color mapping results (with the same color bar) are presented as right inset. Scale bar: 1 µm. b,c) Polarization‐dependent Raman and PL enhancement factor, respectively.

Due to the unique structure of 1D NW, the threefold rotational symmetry of InSe is expected to be broken in the 2D–1D mixed‐dimensional heterostructures, giving rise to fascinating anisotropic photonics.^[^ ^1^, ^38^ ^]^ To prove this, we perform polarization‐dependent Raman measurement on InSe/NW samples. The angle (*θ*) between the polarization of the linearly polarized pump excitation and the direction of the NW axis is varied while collecting the Raman signal. *θ* = 0° (90°) refers that the polarization of the pump light is parallel (perpendicular) to the NW axis. As presented in **Figure** **4**a, the Raman response from InSe/NW is highly polarization‐dependent. When the polarization of the incident pump light is parallel to the NW axis, Raman intensity of InSe/NW heterostructures (≈115 cm^−1^) increases by ≈10.2 times, compared to that of bare InSe. In mark contrast, the Raman EF drops to ≈2.2 times when the polarization of the pump light is perpendicular to the NW axis. Corresponding mappings of EFs are presented in the inset, indicating good uniformity. Polarization‐dependent EF of Raman and PL signals are shown in Figure 4b,c, respectively. The PL EF drops from ≈7.6 to ≈2.4 when the polarization of the pump light is perpendicular to the NW axis. Using the definition of degree of anisotropy ((EF_max_ − EF_min_)/(EF_max_ + EF_min_)), we calculate the degree of anisotropy of Raman signal as ≈65%, and for PL, it is ≈52%. The polarization anisotropy in the Raman and PL responses is induced by the symmetry breaking of InSe in the heterostructures with the NW, which introduces a large 1D dielectric contrast due to the 1D bending topological profile. The polarization anisotropy response is similar to the polarization anisotropy widely observed in NWs.^[^ ^39^, ^40^ ^]^

**Figure 5:**
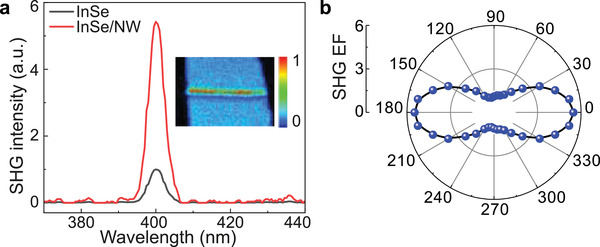
Enhanced nonlinear optical response from the mixed‐dimensional heterostructures. a) Comparison of SHG spectra from the InSe/NW heterostructure and a bare InSe sample. An SHG mapping (normalized to the maximum SHG intensity) is presented as the inset. b) Excitation polarization dependency of SHG enhancement factor.

Apart from Raman and PL responses, we also study the second harmonic nonlinear response of the mixed‐dimensional samples with a home‐built femtosecond laser system. We use linearly polarized excitation at ≈800 nm wavelength and observe the SHG emission peak centered at ≈400 nm. As shown in **Figure** **5**a, when the pump light polarization is parallel to the NW axis (red curve), it is obvious that SHG intensity in the InSe/NW heterostructure significantly enhances (more than fivefolds) compared to that in bare InSe (black curve). Note that no SHG enhancement is observed when the pump wavelength is longer than the InSe PL wavelength (≈1000 nm). Therefore, we can conclude that the SHG enhancement is mainly introduced by the enhanced resonance absorption of the fundamental pump light after the interband transition dipole engineering. Polarization‐resolved EF is shown in Figure 5b. As the polarization of the pump light is rotated to the perpendicular direction to the NW axis (i.e., *θ* = 90°), the EF drops drastically from ≈5.5 times to 1.2 times, further indicating an anisotropic response due to the breaking of the threefold rotational symmetry in the mixed‐dimensional heterostructure.^[^ ^1^, ^3^, ^38^, ^41^, ^42^, ^43^, ^44^ ^]^

Table S1, Supporting Information compares different enhancement methods of optical properties of InSe. Our EF is the highest among the results using the topographical changes, and is comparable to the mechanical strain method. Further, in contrast to the previously demonstrated methods (e.g., strain,^[^ ^31^ ^]^ nanoparticles,^[^ ^32^ ^]^ nanopillar arrays,^[^ ^33^ ^]^ and wrinkles^[^ ^34^ ^]^), our demonstrated robust method offers better uniformity, deterministic positioning and controllability, less fabrication complexity, and larger anisotropic response.

## 3 Conclusion

In conclusion, we have successfully demonstrated 2D–1D mixed‐dimensional heterostructures to engineer the dipole orientation and symmetry breaking of InSe. Benefitting from the enhanced IP dipole moments, both the linear and nonlinear optical responses (e.g., Raman, PL, and SHG) of InSe are enhanced by more than five times with the maximum EF of up to ≈12 in the 2D–1D mixed‐dimensional van der Waals heterostructures. Further, highly anisotropic optical responses are demonstrated, showing a great promise toward various polarization‐dependent optoelectronic applications like photodetectors.

## 4 Experimental Section

### Sample Preparation and Characterization

AlGaAs NWs were grown on silicon substrates inside a horizontal‐flow metalorganic vapor phase epitaxy reactor following Au nanoparticle‐assisted vapor‐liquid‐solid growth method.^[^ ^45^ ^]^ The as‐grown, randomly pointing NWs on parent substrates were mechanically slid over O_2_‐plasma cleaned SiO_2_/Si target substrates to self‐align the NWs in the horizontal plane and pointed toward one direction. 2D InSe/1D NW mixed‐dimensional samples were prepared with commercially available *γ*‐phase InSe crystal (2D semiconductors) following deterministic dry‐transfer method. Finally, samples were annealed in vacuum at 200 °C for an hour. The topology of the fabricated 2D InSe/1D AlGaAs NW devices was analyzed with an atomic force microscope (AFM Dimension Icon, Bruker).

### Raman and PL Measurements

All Raman and PL spectra were acquired at room temperature using a micro‐Raman spectrometer (WITec alpha300) in a confocal backscattering geometry. A frequency‐doubled Nd:YAG solid‐state laser at 532 nm was used as excitation. The linearly polarized incident laser beam was focused perpendicularly (along the *z*‐direction) onto the samples with a spot size of ≈1 µm using an objective lens (100×, 0.9 NA). The backscattered signal was collected through the same objective lens and subsequently dispersed with 1800‐ and 500‐groove mm^−1^ gratings to obtain Raman and PL spectra, respectively, that were detected by a Si‐charge‐coupled camera. Polarization‐resolved measurements were performed by passing the linearly‐polarized excitation laser beam through a half‐wave plate that could tune the polarization angle in the *XY* plane. All experiments were performed at low excitation power (*P* ≤ 0.4 mW) to avoid sample damage and excessive heating.

### SHG Measurements

An ultrafast laser system was employed for the SHG measurements.^[^ ^46^ ^]^ An optical parametric amplifier (TOPAS from Spectra‐Physics) with a repetition rate of 2 kHz was used to generate the light pulses. The width of light pulses was ≈230 fs. The fundamental source of the optical parametric amplifier at ≈800 nm was used to generate SHG at 400 nm. The incident beam was first focused onto the sample with an objective (NA = 0.75, 40×). The spot size of the beam on the sample was ≈3 µm. The reflected light was separated from the input light using appropriate filters and measured with a photomultiplier tube following a monochromator (Andor 328i, Hamamatsu). The SHG mapping of samples was obtained using a home‐build multiphoton microscope system,^[^ ^47^ ^]^ where an ultrafast laser at ≈800 nm with a repetition rate of ≈84 MHz was used. The laser beam was scanned with a 2D galvo mirror system and focused on the sample using an objective lens (20×, 0.4 NA).

## Conflict of Interest

The authors declare no conflict of interest.

## Supporting information

Supporting Information

## Data Availability

The data that support the findings of this study are available from the corresponding author upon reasonable request.
