# Supplementary material for: Engineering the Dipole Orientation and Symmetry Breaking with Mixed‐Dimensional Heterostructures
Source: Adv Sci (Weinh). 2022 May 9;9(20):2200082. doi: 10.1002/advs.202200082 (PMC9284189; doi:10.1002/advs.202200082)
Supplement: Supplementary file 1 — Supporting Information [file ADVS-9-2200082-s001.pdf]

Supporting Information

Engineering the dipole orientation and symmetry breaking with mixed-dimensional heterostructures

*MD Gius Uddin, Susobhan Das, Abde Mayeen Shafi, Vladislav Khayrudinov, Faisal Ahmed, Henry Fernandez, Luojun Du, Harri Lipsanen, Zhipei Sun**

M. G. Uddin, S. Das, A.M. Shafi, V. Khayrudinov, F. Ahmed, H. A. Fernandez, L. Du, Prof. H. Lipsanen, Prof. Z. Sun

Prof. Z. Sun
Department of Electronics and Nanoengineering
Aalto University
Tietotie 3, FI-02150, Finland
E-mail: Zhipei.sun@aalto.fi

Prof. Z. Sun
QTF Centre of Excellence, Department of Applied Physics
Aalto University
Aalto FI-00076, Finland

S1. The vibrational properties of multi-layer InSe in mixed-dimensional heterostructures.

A 532 nm laser excitation is used to investigate the possible strain effect in the mixed-dimensional heterostructures. In the bent InSe region, compared with Raman response from flat InSe, the 176 cm^{-1} and 227 cm^{-1} modes show a shift of -0.78±0.1 cm^{-1} and -0.77±0.1 cm^{-1}, respectively. The previous study[1] on 30-40 layers of InSe under uniaxial tensile strain (produced by the substrate deflection method) reports a shift of -1.5±0.1 cm^{-1}/% for the 176 cm^{-1} Raman mode, and -2.3±0.1 cm^{-1}/% for the 227 cm^{-1} Raman mode. Therefore, we estimate ~ 0.3-0.5% strain in the sample. Nevertheless, with such a small strain, the Raman intensity enhancement is negligible. Therefore, we exclude the strain-induced Raman enhancement in our mixed-dimensional heterostructures.

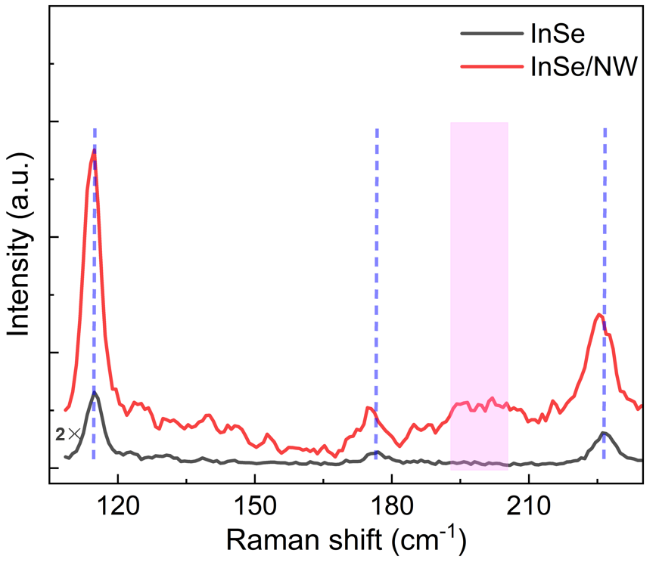

Figure S1: Comparison of InSe Raman spectra -with and -without NW.

S2. PL shift of multi-layer InSe in mixed-dimensional heterostructures.

InSe PL spectra, as shown below, are acquired under 532 nm laser excitation. Solid black and red lines are obtained after fitting the InSe PL data points for the flat and bent regions, respectively. From the fitted curves, we observe a slight redshift (<7 nm, or <9 meV) of PL peak position in the bent InSe region. An earlier study[2] on thick InSe (>30 nm) under tensile strain reports a persistent redshift of ~81 meV/% strain. Therefore, we can estimate a possible strain effect of ~ 0.1% in our mixed-dimensional heterostructures, which is negligible.

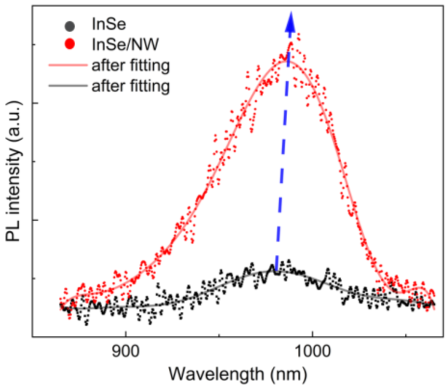

Figure S2: Comparison of InSe PL peak position -with and -without NW.

S3. Assessment of optical interference, substrate, and energy transfer effects in mixed-dimensional heterostructures.

We specially prepare a mixed-dimensional heterostructure sample with a large topographic change to study the optical interference effect. The sample is fabricated by sandwiching a thick hBN flake between InSe and NW. Figure S3(a) presents an optical image of the sample. As shown in Figure S3(b), AFM measurements indicate that thickness of hBN and InSe flakes in the sample is ~40 nm and ~30 nm, respectively. Due to the thick hBN layer underneath, in the InSe/hBN/NW region, the height of the heterostructure area is large (comparable to the PL excitation and emission wavelengths). If the PL enhancement is due to the optical interference effect, we expect to see that the PL enhancement is strongly modulated with the height.

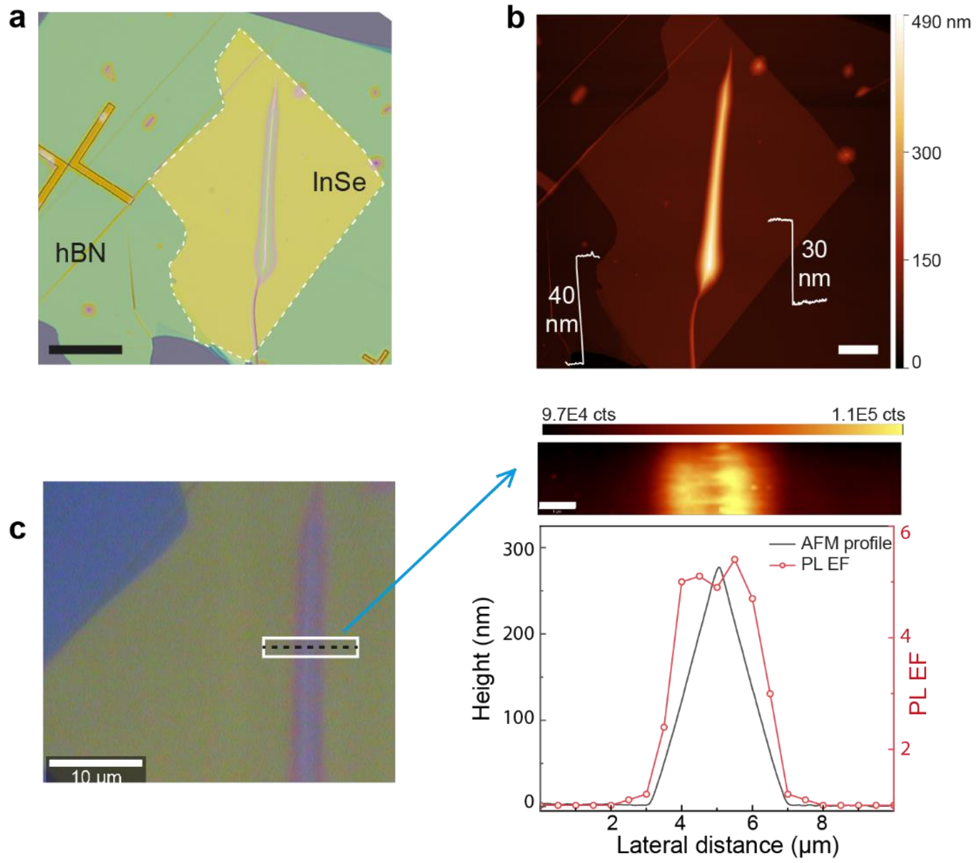

Figure S3: Assessment of optical interference, suspension, and energy transfer effects in mixed-dimensional heterostructures. (a) Optical image of a thick hBN sandwiched (between InSe and NW) device. A dashed white line indicates the edge of the InSe flake. Scale bar: 20 μm. (b) AFM topography of the sample along with hBN (~40 nm) and InSe (~30 nm) thickness profiles. Scale bar: 10 μm. (c) The rectangular white box in the optical image indicates the area of the PL scan, and the black dashed line inside the box indicates the path of the AFM line scan. The corresponding PL map is presented on the top right side of the panel. The correlation between the AFM height profile and PL enhancement factor (EF) is presented on the bottom right side, which can exclude interference, substrate effect, and energy transfer as reasons behind enhanced optical properties in our proposed mixed-dimensional heterostructures.

We perform PL measurements on the sample and correlate the results with corresponding AFM topography. The white box in Figure S3(c) shows a PL scan area of 10 μm × 2 μm. The black dashed line in the white box indicates the path of the AFM line scan. The height of the topologically manipulated InSe layer in the heterostructure is measured as ~270 nm. However, in the PL enhancement results (red lines in the bottom right figure of Figure S3(c)), we do not observe any optical interference effect (e.g., enhancement factor modulation as a function of the height in such a large topologic change). Therefore, we can rule out the optical

interference effect as the reason behind enhanced optical properties in our proposed mixed-dimensional heterostructures.

In the specially-prepared heterostructure sample, the lateral length of the bending region (i.e., suspension region) is also very large (~4 μm, see the AFM results in the bottom right figure of Figure S3(c)), much larger than the diameter of the NW of ~270 nm. If the PL enhancement is due to the sample suspension, we would expect to see that the PL enhancement factor should be quite uniform in the suspension region. However, in the bending suspension region, the enhancement factor increases significantly (e.g., at the lateral distance of ~3 μm) and starts to saturate (e.g., at the lateral distance of ~3.5 μm). Therefore, we can rule out the substrate and suspension effects as the reason behind enhanced optical properties in our proposed mixed-dimensional heterostructures.

Note that this specially prepared device also confirms that the PL enhancement is not because of the energy transfer effect, as we start to see the PL enhancement in the bending area (e.g., at the lateral distance of ~2.5 μm) that is far away from the NW position (at the lateral distance of ~5 μm), as shown in the bottom right figure of Figure S3(c).

S4. Angle-resolved SHG response of mixed-dimensional heterostructures.

The six-fold symmetry of SHG in InSe (black curve in Figure S4) is similar to the previous studies[3-5]. Interestingly, the enhanced SHG emission from the heterostructure region (red curve) no longer follows the six-fold symmetry pattern, indicating symmetry breaking in the InSe/NW heterostructure compared to the bare InSe sample.

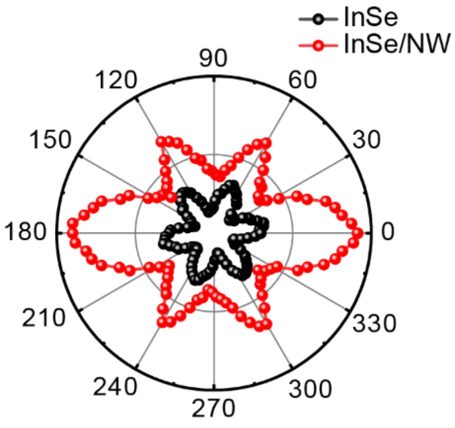

Figure S4: Comparison of polar plots of SHG intensity of InSe as a function of the rotation angle in a “parallel” configuration. During the measurements, first, the initial polarization of

the incident light and the polarization of the analyzer are set along the NW axis. Afterwards, both the polarizer and the analyzer were rotated together from 0° to 360° degrees in a step of 4°.

S5. Efficacy of mixed-dimensional heterostructure approach to tune optical properties of InSe.

Table S1: Summarized comparison of enhancement methods of optical properties of InSe.

Modulation strategy	Method/Substrate	InSe thickness	Maximum Enhancement (Raman, PL, SHG)	Reference
Strain	Mechanical bending with PETG substrates (with a uniaxial strain of up to ~1.15%)	10–15 layers	18±4 times (A_1 Raman mode at 199 cm^{-1})	[1]
Topographical change	SiO_2 nanoparticles	~12 nm	~5 times (PL)	[6]
	Mechanically exfoliated wrinkles on SiO_2/Si substrates	~80 nm	~3 times (PL)	[7]
	Si nanopillar array	> 20 nm	~3 times (PL, Raman)	[8]
	Integration with AlGaAs NWs	~35 nm	~12 times (A_1 Raman mode at 115 cm^{-1})	Our work
			~9 times (PL)	
			~5.5 times (SHG)	

References

[1] C. Song, F. Fan, N. Xuan, S. Huang, C. Wang, G. Zhang, F. Wang, Q. Xing, Y. Lei, Z. Sun, H. Wu, H. Yan, *Phys. Rev. B* **2019**, *99*, 195414.

[2] Y. Li, T. Wang, M. Wu, Ting Cao, Y. Chen, R. Sankar ,R. K. Ulaganathan, F. Chou, C. Wetzel, C. Xu, S. G. Louie, S. Shi, *2D Mater.* **2018**, *5*, 021002.

[3] S. Deckoff-Jones, J. Zhang, C. E. Petoukhoff, M. Man, S. Lei, R. Vajtai, P. M. Ajayan, D. Talbayev, J. Madéo, K. M. Dani, *Sci. Rep.* **2016**, *6*, 22620.

[4] Q. Hao, H. Yi, H. Su, B. Wei, Z. Wang, Z. Lao, Y. Chai, Z. Wang, C. Jin, J. Dai, W. Zhang, *Nano Lett.* **2019**, *19*, 2634.

[5] N. Leisgang, J. G. Roch, G. Froehlicher, M. Hamer, D. Terry, R. Gorbachev, R. J. Warburton, AIP Adv. **2018**, 8, 105120.

[6] M. B. Gisbert, D. A. Penares, J. Suh, F. Hidalgo, R. Abargues, P. J. Cantó, A. Segura, A. Cros, G. Tobias, E. Canadell, P. Ordejón, J. Wu, J. P. Pastor, J. F. Royo, Nano Lett. **2016**, 16, 3221
[7] Y. Li, T. Wang, H. Wang, Z. Li, Y. Chen, D. West, R. Sankar, R. K. Ulaganathan, F Chou, C. Wetzel, C.Xu, S. Zhang, S. Shi, Nano Lett. **2018**, 18, 5078.
[8] D. Mazumder, J. Xie, Z. R. Kudrynskyi, X. Wang, O. Makarovsky, M.A. Bhuiyan, H. Kim, T. Chang, D. L. Huffaker, Z. D. Kovalyuk, L. Zhang, A. Patanè, Adv. Optical Mater. **2020**, 8, 2000828.
